# Weight Loss After Laparoscopic Sleeve Gastrectomy Ameliorates the Cardiac Remodeling in Obese Chinese

**DOI:** 10.3389/fendo.2021.799537

**Published:** 2022-01-21

**Authors:** Weilun Meng, Ronggang Peng, Lei Du, Yixing Zheng, Diya Liu, Shen Qu, Yawei Xu, Yi Zhang

**Affiliations:** ^1^ Department of Cardiology, Shanghai Tenth People’s Hospital, Nanjing Medical University, Shanghai, China; ^2^ Department of Cardiology, Affiliated Hospital of Jiangnan University, Wuxi, China; ^3^ Department of Metabolic Surgery, Shanghai Tenth People’s Hospital, Tongji University, Shanghai, China; ^4^ Department of Thyroid and Breast Surgery, Shanghai Tenth People’s Hospital, Tongji University School of Medicine, Shanghai, China; ^5^ Department of Endocrinology and Metabolism, Shanghai Tenth People’s Hospital, Tongji University, Shanghai, China; ^6^ Department of Cardiology, Shanghai Tenth People’s Hospital, Tongji University School of Medicine, Shanghai, China

**Keywords:** sleeve gastrectomy, cardiovascular, obesity, echocardiography, cardiac structural and functional remodeling

## Abstract

This study aimed to investigate the impact of weight loss after laparoscopic sleeve gastrectomy (LSG) on cardiac structural and functional remodeling in obese Chinese. A total of 44 obese participants were enrolled consecutively. The physical, laboratory, electrocardiographic, and echocardiographic parameters of pre-and postoperative were recorded. The average follow-up time was 12.28 ± 5.80 months. The body mass index (BMI) of the patients with obesity was decreased from 41.6 ± 7.44 to 30.3 ± 5.73kg/m^2^ (*P*<0.001) after LSG. The systolic and diastolic blood pressure of the subjects was significantly reduced from 137.9 ± 15.7mmHg to 123.0 ± 16.0 and 83.4 ± 10.8 to 71.3 ± 11.7mmHg (P<0.001), respectively. The levels of fasting insulin and fasting blood glucose were significantly decreased (38.8 ± 32.1 to 8.43 ± 4.16 mU/L, *P*<0.001; 6.95 ± 2.59 to 4.64 ± 0.50mmol/L, *P*<0.001). Total cholesterol (TC, 4.66 ± 0.84 to 4.23 ± 0.75mmol/L, P<0.001) and triglyceride (TG, 1.92 ± 1.21 to 0.85 ± 0.30mmol/L, P<0.001) decreased significantly. Cardiovascular geometric parameters including aortic sinus diameter (ASD, 32.9 ± 2.83mm to 32.0 ± 3.10mm, P<0.05), left atrial diameter (LAD, 38.8 ± 4.03 to 36.2 ± 4.12mm, P<0.001), and interventricular septum thickness(IVS, 10.2 ± 0.93 to 9.64 ± 0.89mm, P<0.001) were significantly reduced. The ratio of weight loss (RWL) was positively correlated with the changes of LAD. The change of IVS was negatively correlated with the change of fasting blood glucose (GLU). Weight loss after LSG could effectively improve cardiac structural, but not functional, abnormality in obese Chinese.

## Introduction

Obesity refers to the excessive accumulation of fat that is harmful to health and the body mass index (BMI) is more than 30kg/m^2^ caused by the imbalance of energy metabolism, which is the most common chronic metabolic disease. According to statistics, the number of obese people in the world exceeded 650 million in 2016, and the prevalence is on the rise (1, 2). Obesity is one of the risk factors for cardiovascular disease, which can cause long-term damage to the cardiovascular structure and function (3, 4). The traditional treatment of obesity mainly includes diet control, exercise, medication and so on, but the effect is not satisfactory (5). Bariatric surgery is the only sustained and effective treatment for obesity as a surgical pattern (6). It has been proved that weight loss by bariatric surgery such as laparoscopic sleeve gastrectomy (LSG) could significantly improve the energy metabolism of the patients with obesity, reduce their cardiac lipotoxicity, and play a more extensive role than simply losing weight, so as to ameliorate the cardiac structure and function (5, 7). However, the study of the effect of weight loss after LSG on the cardiac is not yet available in the Chinese population, and this protocol is to study the effects of LSG on the cardiac structure and function in obese Chinese.

## Materials and Methods

**Figure 1:**
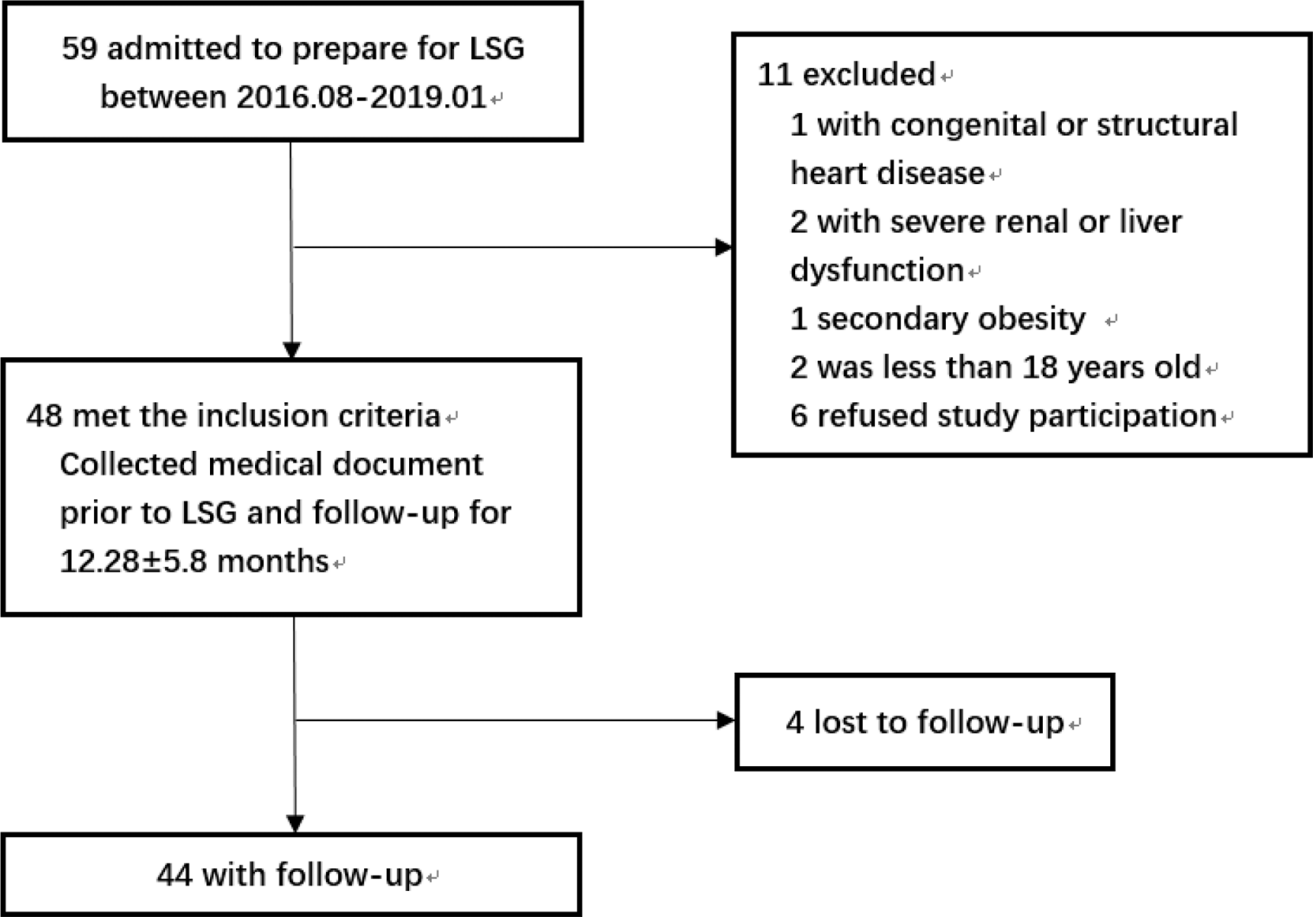
Flowchart of the study. LSG, laparoscopic sleeve gastrectomy.

The flowchart of the study was shown in **Figure 1**. The study included patients with obesity admitted to the hospital who were preparing for LSG from August 2016 to January 2019 and met the indications: BMI≥ 35kg/m^2^ or BMI≥ 27.5kg/m^2^ with other comorbidities (8). The exclusion criteria were as follows: patients with congenital heart disease or structural cardiomyopathy; patients with severe renal dysfunction or liver dysfunction; secondary obesity such as Cushing’s syndrome, hypothyroidism, or taking drugs that affected body weight; The age was less than 18 years old; refused to participate in this study. The surgery procedures of all participants were performed by the same team. The Ethical Committee of the hospital approved this study and all the subjects volunteered to participate in this study by signing informed consent.

### Medical History and Physical Measurements

Detailed medical history was acquired by a well-trained physician, including smoking, hypertension (HTN), and the use of antihypertensive drugs. The operation time and follow-up time were recorded. The BMI was calculated by the formula: weight/height^2^. The ratio of weight loss (RWL) was defined as the change of body weight dividing the preoperative weight. The systolic blood pressure (SBP) and diastolic blood pressure (DBP) of the patients with obesity was measured according to the guideline (9).

### Laboratory Examinations

The subjects’ fasting venous blood serum samples were taken before operation and at the follow-up to test related parameters, including low density lipoprotein (LDL), total cholesterol (TC), triglyceride (TG), high density lipoprotein (HDL), free fatty acid (FA), fasting blood glucose (GLU), fasting insulin (Ins) and glycosylated hemoglobin A1c (HbA1c).

### Electrocardiogram Examination

The subjects were required to take a standard resting electrocardiogram examination by a trained physician using the Cardiofax S ECG machine (NIHON KOHDEN, Japan). A 10s 12-leads synchronous electrocardiogram was recorded, with a paper speed of 25 mm/s and an amplitude of 10 mm/mV. HR was measured according to the RR interval on ECG. PR referred to the interval from the start of the P wave to the start of QRS complexes. The corrected QT interval (QTc) was calculated using Bazett’s formula: QT/RR^1/2^.

### Echocardiography Examinations

Echocardiography was blindly performed by an experienced echocardiographer using a MyLab 30 cardiovascular machine (ESAOTE SPA) with a 3.5MHz probe, according to the American Society of Echocardiography recommendations (10). The aortic sinus diameter (ASD), left atrial diameter (LAD), left ventricular end-diastolic diameter (LVEDD), left ventricular end-systolic diameter (LVESD), interventricular septum thickness (IVS), and left ventricular posterior wall thickness (PWT) were measured by M-mode. Left ventricle mass (LVmass) was estimated by the design formula:0.8*(1.04*(LVEDD+IVS+PWT)^3^)+0.6 (11). The peak of systolic mitral annular velocities (Sa) was measured by Doppler tissue imaging while the LVEF was measured by M-mode Teichholz.

### Statistical Analysis

All statistical analyses were performed using the SPSS20.0 software (IBM Corp., USA). All continuous variables were presented as mean ± SD and categorical variables were presented as percentages. The changes of parameters before and after LSG were represented by Δ. The continuous indexes of preoperative and postoperative were compared by paired sample t-test and the percentages by the chi-square test. The bivariate relationship between the cardiac parameters and cardiovascular risk factors was studied by Pearson correlation analysis. Multiple linear regression analysis was used to assess the correlation between cardiac parameters and cardiovascular risk factors. The hypothesis testing can be considered to be of statistical significance when *P <*0.05.

## Results

A total of 44 subjects participated in the study, including 18 men and 26 women. The mean age of the subjects was 32.4 ± 9.6 years old, and the average follow-up time was 12.28 ± 5.80 months. The proportion of subjects taking antihypertensive drugs was 18% and that of smokers was 9%. The BMI of the patients with obesity after LSG decreased significantly from 41.6 ± 7.44 to 30.3 ± 5.73kg/m^2^ (*P*<0.001). 11 patients had hypertension (HTN) diagnoses 3 [1-20] years before the LSG, 8 of which have taken the antihypertensive drugs. The proportion of subjects who had taken the antihypertensive drugs would be reduced (18.2 to 6.8%, P<0.05) and the types of antihypertensive drugs also reduced (2.5 [1-5] to 0 [0-3], P<0.05) after LSG. Meanwhile, the systolic and diastolic blood pressure of the subjects who had not taken the antihypertensive drug was reduced (137.9 ± 15.7 to 123.0 ± 16.0mmHg, *P*<0.001; 83.4 ± 10.8 to 71.3 ± 11.7mmHg, *P*<0.001). The levels of fasting insulin and fasting blood glucose were significantly decreased (38.8 ± 32.1 to 8.43 ± 4.16 mU/L, *P*<0.001; 6.95 ± 2.59 to 4.64 ± 0.50mmol/L, *P*<0.001), and HbA1c decreased from 7.10 ± 2.39 to 5.50 ± 0.37% (*P*<0.001). LDL (2.86 ± 0.74 to 2.59 ± 0.76mmol/L, P=0.005), TC (4.66 ± 0.84 to 4.23 ± 0.75mmol/L, P<0.001), TG (1.92 ± 1.21 to 0.85 ± 0.30mmol/L, P<0.001) and HDL (1.02 ± 0.21 to 1.23 ± 0.26mmol/L, P<0.001) have been improved to a certain extent, relatively. However, FA failed to reach a statistically significant decrease. The basic characteristics and the changes of conventional cardiovascular risk factors and metabolism variables at baseline and follow-up were described in **Table 1**.

**Table 1:** Baseline characteristics, conventional cardiovascular risk factors, and metabolism variables at baseline and follow-up.

	Baseline	12-month Follow-up	*P* value
Age (years)	32.4 ± 9.6		
Male, n (%)	18 (41%)		
Cigarette smoking, n (%)	4 (9%)	4 (9%)	1
BMI (kg/m2)	41.6 ± 7.44	30.3 ± 5.73	<0.001
SBP (mmHg)	137.9 ± 15.7	123.0 ± 16.0	<0.001
DBP (mmHg)	83.4 ± 10.8	71.3 ± 11.7	<0.001
LDL (mmol/L)	2.86 ± 0.74	2.59 ± 0.76	0.014
GLU (mmol/L)	6.95 ± 2.59	4.64 ± 0.50	<0.001
TC (mmol/L)	4.66 ± 0.84	4.23 ± 0.75	0.005
TG (mmol/L)	1.92 ± 1.21	0.85 ± 0.30	<0.001
HDL (mmol/L)	1.02 ± 0.21	1.23 ± 0.26	<0.001
FA (mmol/L)	0.57 ± 0.22	0.52 ± 0.21	0.28
Ins (mU/L)	38.8 ± 32.1	8.43 ± 4.16	<0.001
HbA1c (%)	7.10 ± 2.39	5.50 ± 0.37	<0.001
HTN	11 (25.0%)	3 (6.8%)	<0.05
Years of HTN	3 [1-20]		
Antihypertensive drugs taking, n (%)	8 (18.2%)	3 (6.8%)	<0.05
Types of antihypertensive drugs	2.5 [1-5]	0 [0-3]	<0.05

Data were presented as absolute number (%) or mean ± SD. Student’s T-test and chi-square test.

were conducted to compare the differences between baseline and 12-month follow-up for quantitative and qualitative variables, respectively. BMI, body mass index; SBP, systolic blood pressure; DBP, diastolic blood pressure; LDL, low density lipoprotein; GLU, fasting blood glucose; TC, total cholesterol; TG, triglyceride; HDL, high density lipoprotein; FA , free fatty acid; Ins, fasting insulin; HbA1c, glycosylated hemoglobin A1c; HTN, hypertension.

**Figure 2:**
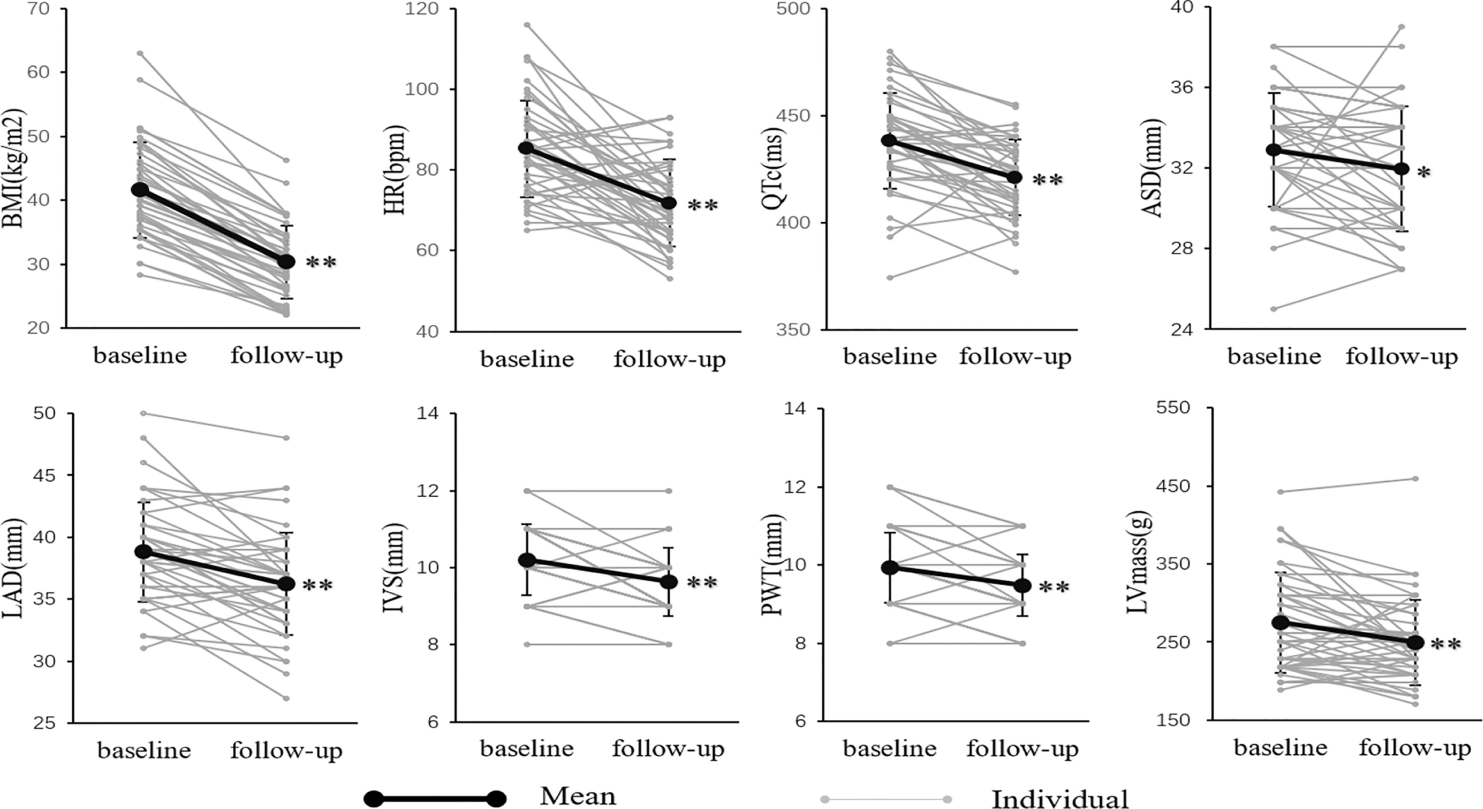
The BMI, electrocardiographic and echocardiographic parameters at baseline and follow-up. *P < 0.05; **P < 0.01. There was a significant decrease in anthropometric, electrocardiographic, and echocardiographic parameters, including BMI, HR, QTc, ASD, LAD, IVS, PWT, and LVmass.

The changes in electrocardiographic and echocardiographic parameters were summarized in **Table 2**. The heart rate (HR) decreased from 85.2 ± 12.2 to 41.91 ± 10.85 beats/minute (*P*<0.001) after LSG. There was no significant change in PR interval. In one of the subjects, who was treated with a β-receptor blocker for a long time, the QTc significantly extended to 619 ms, which met the diagnostic criteria of long QT interval syndrome. Excluding this outlier value, QTc significantly shortened from 438.1 ± 22.3 to 421.0 ± 17.6ms (*P*<0.001). The subjects had a significant reduction in ASD and LAD after weight loss (32.9 ± 2.83 to 32.0 ± 3.10mm, *P*<0.05; 38.8 ± 4.03 to 36.2 ± 4.12mm, *P*<0.001), while LVEDD and LVESD did not change significantly. The subject’s IVS and PWT were thinner than before surgery (10.2 ± 0.93 to 9.64 ± 0.89mm, *P*<0.001; 9.93 ± 0.90 to 9.48 ± 0.79mm, *P*<0.001). The LVmass was reduced from 275.0 ± 64.2 to 249.7 ± 54.3g(*P*<0.05). However, the LVEF and Sa had no significant change. These changes in changes in BMI, cardiac electrophysiological, structural, and functional parameters were shown in **Figure 2** more clearly.

**Table 2:** Electrocardiographic and echocardiographic parameters at baseline and follow-up.

	Baseline	Follow-up	*P* value
Electrocardiogram			
HR (beats/minute)	85.2 ± 12.2	71.9 ± 10.9	<0.001
PR interval (ms)	153.5 ± 13.7	155.3 ± 14.1	0.34
QTc (ms)	438.1 ± 22.3	421.0 ± 17.6	<0.001
Echocardiogram			
ASD (mm)	32.9 ± 2.83	32.0 ± 3.10	0.020
LAD (mm)	38.8 ± 4.03	36.2 ± 4.12	<0.001
LVEDD (mm)	48.6 ± 4.41	47.5 ± 3.94	0.087
LVESD (mm)	30.4 ± 5.01	29.9 ± 3.80	0.41
IVS (mm)	10.2 ± 0.93	9.64 ± 0.89	<0.001
PWT (mm)	9.93 ± 0.90	9.48 ± 0.79	<0.001
LVmass (g)	275.0 ± 64.2	249.7 ± 54.3	0.001
LVEF (%)	64.2 ± 7.26	65.4 ± 3.53	0.28
Sa (m/s)	0.10 ± 0.03	0.10 ± 0.02	0.88

Data were presented as absolute number (%) or mean ± SD. Student’s t-test and chi-square test were conducted to compare the differences between baseline and 12-month follow-up for quantitative and qualitative variables, respectively. HR, heart rate; QTc, corrected QT interval; calculated by Bazett’s formula: QT/RR1/2; ASD, aortic sinus diameter; LAD, left atrial diameter; LVEDD, left ventricular end-diastolic diameter; LVESD, left ventricular end-systolic diameter; IVS, interventricular septum thickness; PWT, left ventricular posterior wall thickness; LVmass, left ventricular mass; calculated by the formula:0.8* (1.04 * (LVEDD+IVS+PWT)* 3)*0.6); LVEF, left ventricular ejection fraction; Sa, The peak of systolic mitral annular velocities.

**Table 3** showed the correlations between conventional cardiovascular risk factors and the cardiac parameters. **Table 4** showed the results of multiple linear regression models that were used to assess the independent association between conventional cardiovascular risk factors and cardiac parameters. The changes of LAD were significantly positively correlated with RWL and the changes of IVS were negatively correlated with the changes of GLU. And after adjustment for age and gender, the independent association was still tenable.

**Table 3:** Correlations of conventional cardiovascular risk factors and the cardiac parameters.

	ΔHR		ΔQTc		ΔLAD		ΔIVS		ΔPWT	
	R	P	R	P	R	P	R	P	R	P
RWL	0.057	0.71	-0.068	0.66	0.470**	0.001	-0.005	0.97	-0.17	0.27
ΔSBP	-0.18	0.25	-0.036	0.82	0.14	0.35	-0.018	0.91	0.19	0.23
ΔLDL	-0.034	0.83	-0.11	0.49	0.057	0.72	-0.12	0.43	-0.25	0.10
ΔGLU	0.070	0.65	0.15	0.34	0.093	0.55	-0.34*	0.024	-0.37*	0.015

Pearson correlation analyses were performed to compare the coefficients. *P < 0.05; **P < 0.01. HR, heart rate; QTc, corrected QT interval; calculated by Bazett’s formula: QT/RR1/2; LAD, left atrial diameter; IVS, interventricular septum thickness; PWT, left ventricular posterior wall thickness; RWL, ratio of weight loss; SBP, systolic blood pressure; LDL, low density lipoprotein; GLU, fasting blood glucose. The changes of parameters pre-and postoperative were represented by Δ.

**Table 4:** Multiple linear regression analysis of conventional cardiovascular risk factors and cardiac parameters.

Variables included in the model	ΔLAD		ΔIVS		ΔPWT		
	β	P	β	P	β	P	
Without adjustment							
RWL	0.50**	0.001	0.021	0.90	-0.13	0.39	
ΔSBP	0.15	0.31	-0.081	0.62	0.20	0.20	
ΔLDL	-0.16	0.30	-0.059	0.73	-0.22	0.17	
ΔGLU	0.15	0.30	-0.35*	0.031	-0.29	0.055	
With adjustment for age and gender								
RWL	0.50**	0.002	0.021	0.90	-0.12	0.43	
ΔSBP	0.14	0.39	-0.072	0.68	-0.18	0.29	
ΔLDL	-0.15	0.34	-0.069	0.69	-0.24	0.15	
ΔGLU	0.14	0.37	-0.33*	0.047	-0.29	0.065	

Association of conventional cardiovascular risk factors and the parameters of cardiac was analyzed by the multivariable linear regression analysis with and without adjustment for age and gender. *P < 0.05; **P < 0.01. LAD, left atrial diameter; IVS, interventricular septum thickness; PWT, left ventricular posterior wall thickness; RWL, ratio of weight loss; SBP, systolic blood pressure; LDL, low density lipoprotein; GLU, fasting blood glucose. The changes of parameters pre-and postoperative were represented by Δ.

**Figure 3:**
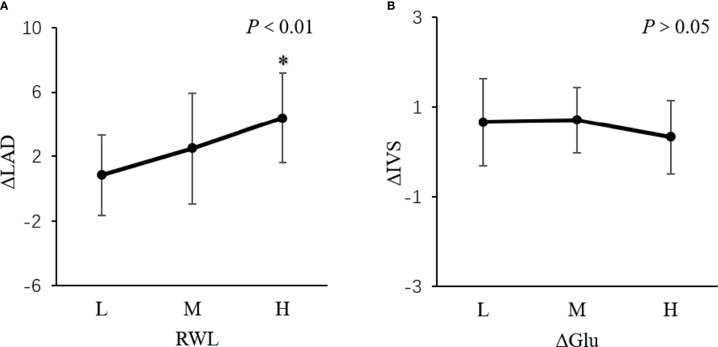
The change of LAD and IVS in subgroups. *P < 0.01. The population was divided into low (L), middle (M), and high (H) subgroups according to the tri-sectional quantiles of weight loss ratio (RWL) or the change of glucose (ΔGLU). **(A)** showed the change of LAD was different among the subgroups (P <0.01). But **(B)** showed the change of IVS was not different in subgroups (P > 0.05).

The population was divided into low (L), middle (M), and high (H) subgroups according to the tri-sectional quantiles of RWL or the change of GLU. Among the subgroups, the change of LAD was different (*P*<0.01, **Figure 3A**). But the change of IVS was not different in subgroups (*P*>0.05, **Figure 3B**).

## Discussion

In this study, we found that weight loss after LSG could reduce cardiovascular risk factors and ameliorate the cardiac structure, but failed to show a significant improvement in cardiac function. In addition, the electrical activity of cardiomyocytes also had a beneficial change, with the QTc interval moderately shortened after the operation. Besides, we found that there was a positive correlation between LAD and RWL in patients with obesity after successful slimming, while the change of IVS was negatively correlated with the change of GLU. To our best knowledge, we are the first to study the effect of LSG on cardiac structure and electrical activity.

The metabolic disorder of cardiomyocytes in patients with obesity leads to cardiomyocyte steatosis, and then apoptosis and fibrosis, causing ventricular remodeling at the cellular and tissue levels (12, 13). On the other hand, hemodynamic changes in patients with obesity give rise to cardiac structural remodeling (14, 15), such as ventricular hypertrophy, left atrial dilatation, etc. (5) Several studies have shown that the left ventricular volume of patients with obesity decreased significantly at 3-36 months after weight loss (16–18). Similarly, our study confirmed that patients with obesity decreased after weight loss, but the left ventricular diameter did not change significantly. Mukerji R, et al. (19) suggested that only left ventricular with hypertrophy could significantly reduce the volume after weight loss, but not in patients without hypertrophy. The left ventricular hypertrophy of the subjects in our study was mild, so there may be no significant change in the left ventricle diameter. A meta-analysis (17) showed that left ventricular mass and interventricular septum decreased significantly after weight loss. Our study also confirmed that left ventricular mass decreased 25.32 ± 49.40g, and interventricular septum and left ventricular posterior wall thinned significantly. In experiments with mice as a model, Zhang X, et al. (20) showed that endoplasmic reticulum stress-mediated cardiomyocytes apoptosis could be alleviated and cardiomyocytes arranged more neatly and denser after weight loss. And Huang X, et al. (21) demonstrated that weight loss could promote intracellular Ca^2+^ homeostasis and extenuate myocardial autophagy. The reverse of cardiac cells and tissue may refer to the above mechanism. In addition, weight loss can reduce the pre-, post-load, and pulmonary artery pressure in the heart of the obese patient, thereby ameliorating the remodeling of the ventricular structure (22–26).

Obesity can cause left ventricular diastolic and systolic dysfunction, A meta-analysis (17) showed that the cardiac systolic function of the obesity was improved after weight loss. Nevertheless, several studies also suggested that the LVEF did not change significantly (16, 18). McCloskey CA, et al. (27) found that LVEF increased significantly after weight loss in patients with obesity with LVEF < 40%, suggesting that the change of LVEF may be different in patients with obesity with preserved and reduced left ventricular systolic function. A number of studies have demonstrated a significant improvement in the diastolic function of the heart in patients with obesity after weight loss (16–18, 27). In addition, the ratio of E/A in the patients with normal heart function was significantly improved after weight loss, suggesting that the pseudo-normal heart function was improved after weight loss (16, 18). In our study, the changes in left ventricular ejection fraction and left ventricular diastolic function were not significant, possibly due to the enrolled patients were so young and with little degeneration of the heart function.

Up to 29% of severe patients with obesity had a QTc interval of more than 440ms, which meant abnormal ventricular repolarization and increased the risk of sudden cardiac death (28).The weight loss especially caused by bariatric surgery has been proved to shorten the Qtc interval (28, 29). Our study also confirmed that the QTc interval was moderately shortened after weight loss. Mukerji R, et al. (19) showed that the significant shortening of QTc with weight loss occurred only in patients with left ventricular hypertrophy. The mechanism of shortening QTc interval due to weight loss is still unclear, which may be related to the changes in insulin level and other metabolic variables after weight loss (30).

Through the correlation analysis, we found that the change of left atrial diameter was significantly associated with the proportion of body weight change, independent of the improvement of blood pressure, which was also consistent with a former study (31). We also found that there was a negative correlation between the changes in the ventricular wall thickness and the changes in blood glucose levels. The reason may be that the cardiomyocytes were in a catabolic state after LSG, and it can also partly explain the reasons for the decline in cardiac mass.

Besides weight loss, the effect of the hormones change may indeed play an important role in cardiac remodeling after LSG. Unfortunately, we didn’t collect data on hormones change to study the relationship between them and cardiac remodeling. Pilar Cobeta et al. in 2020 have reported the change of hormones in 20 patients 6 months after LSG and demonstrated sex hormone-binding globulin and testosterone reduced 6 months later and they were associated with decrease in carotid intima-media thickness (32). And other hormones such as gastric hormones, intestinal hormones, and pancreatic hormones have been proved to improve after bariatric surgery (33). However, it needs more research to verify their effect on cardiac remodeling.

Our study has several limitations. First, the size of the sample is small and non-random, and the power of the main result statistics is limited. Second, our study is lack of the control group, such as patients on diet or drug and patients who have undergone another bariatric surgery. However, non-surgical methods were difficult to produce sustained weight loss in severely obese people and therefore matched to adequate populations as controls. And other bariatric surgeries have been launched finitely. Third, cardiac benefit after bariatric may be correlated with duration of weight loss, while our follow-up time for patients is not consistent, from 3 months to 24 months. So further research is still needed to clarify the intensity of cardiac benefit at different time nodes.

## Conclusion

Weight loss after LSG could effectively improve cardiac structural, but not functional remodeling in obese Chinese.

## Data Availability Statement

The raw data supporting the conclusions of this article will be made available by the authors, without undue reservation.

## Ethics Statement

This study is in accordance with the ethical standards as laid down in the 1964 Declaration of Helsinki and its later amendments or comparable ethical standards. Written informed consent was obtained from the individual(s) for the publication of any potentially identifiable images or data included in this article.

## Author Contributions

YX and YZha designed the article. WM and RP drafted and revised the manuscript under the supervision of YZha. LD collected basic information of participants. DL, YZhe, and SQ contributed to the manuscript editing. All authors contributed to the article and approved the submitted version.

## Conflict of Interest

The authors declare that the research was conducted in the absence of any commercial or financial relationships that could be construed as a potential conflict of interest.

## Publisher’s Note

All claims expressed in this article are solely those of the authors and do not necessarily represent those of their affiliated organizations, or those of the publisher, the editors and the reviewers. Any product that may be evaluated in this article, or claim that may be made by its manufacturer, is not guaranteed or endorsed by the publisher.
